# Taxonomic Separation of Hippocampal Networks: Principal Cell Populations and Adult Neurogenesis

**DOI:** 10.3389/fnana.2016.00022

**Published:** 2016-03-09

**Authors:** R. Maarten van Dijk, Shih-Hui Huang, Lutz Slomianka, Irmgard Amrein

**Affiliations:** ^1^Functional Neuroanatomy, Institute of Anatomy, University of ZürichZurich, Switzerland; ^2^Neuroscience Center Zurich, University of Zurich and ETH ZurichZürich, Switzerland; ^3^Department of Health Sciences and Technology, Institute of Human Movement Sciences and Sport, ETH ZurichZürich, Switzerland

**Keywords:** correspondence analysis, primates, rodent, stereology, hilus, CA3, CA1, comparative

## Abstract

While many differences in hippocampal anatomy have been described between species, it is typically not clear if they are specific to a particular species and related to functional requirements or if they are shared by species of larger taxonomic units. Without such information, it is difficult to infer how anatomical differences may impact on hippocampal function, because multiple taxonomic levels need to be considered to associate behavioral and anatomical changes. To provide information on anatomical changes within and across taxonomic ranks, we present a quantitative assessment of hippocampal principal cell populations in 20 species or strain groups, with emphasis on rodents, the taxonomic group that provides most animals used in laboratory research. Of special interest is the importance of adult hippocampal neurogenesis (AHN) in species-specific adaptations relative to other cell populations. Correspondence analysis of cell numbers shows that across taxonomic units, phylogenetically related species cluster together, sharing similar proportions of principal cell populations. CA3 and hilus are strong separators that place rodent species into a tight cluster based on their relatively large CA3 and small hilus while non-rodent species (including humans and non-human primates) are placed on the opposite side of the spectrum. Hilus and CA3 are also separators within rodents, with a very large CA3 and rather small hilar cell populations separating mole-rats from other rodents that, in turn, are separated from each other by smaller changes in the proportions of CA1 and granule cells. When adult neurogenesis is included, the relatively small populations of young neurons, proliferating cells and hilar neurons become main drivers of taxonomic separation within rodents. The observations provide challenges to the computational modeling of hippocampal function, suggest differences in the organization of hippocampal information streams in rodent and non-rodent species, and support emerging concepts of functional and structural interactions between CA3 and the dentate gyrus.

## Introduction

The highly laminar organization of the mammalian hippocampus has not only facilitated countless physiological and anatomical studies but also made it relatively easy to recognize if observations made in one species diverged from those in another. Individual hippocampal principal cell populations (granule, hilar, CA3, CA1, or subicular cells) have been found to differ in their cytoarchitectural appearance from species to species (Rosene and Van Hoesen, 1987; Slomianka et al., 2011). Similar differences between species have been found in neurochemical signatures (Gall, 1990; McNamara et al., 1996; Blackshaw et al., 2003; Seress et al., 2004, 2008; Smits et al., 2004; Slomianka et al., 2011) and the distribution of intrahippocampal efferents (Laurberg and Zimmer, 1980; Gaarskjaer et al., 1982; Amaral et al., 1984; van Groen and Wyss, 1988). Extrahippocampal afferents of these cell populations also show species differences in their neurochemical (Murakawa and Kosaka, 1999) and genomic (Mashiko et al., 2012) signatures and their projection patterns to the hippocampus (Schwerdtfeger, 1984; van Groen et al., 2002). Because of the mainly qualitative documentation of species differences, it is difficult to incorporate them into computational models of hippocampal function that may reveal their functional impact. Although species differences vividly illustrate the potential for adaptive change, there is, consequently, rarely evidence for their specific adaptive value. This is furthermore hampered by the small number of phylogenetically disjoint species that have been studied, which makes it difficult to judge if the presumed adaptive value should be looked for in the specific species that has been studied or if it may also be found in other taxonomically and/or behaviorally related species.

The volumes of the hippocampus and its subfields are exceptions to the lack of quantitative data in a large species sample. A textbook finding is the apparent expansion of CA1 with primate evolution (Stephan, 1983; Seress, 1988; West, 1990), but even in this case, the structure-function relationship remains elusive. The allometrically progressive development of the hippocampus from insectivores to primates, and larger than expected progression of CA1 in the human brain (Stephan, 1983), relates the expected size of CA1 to body weight. If the size of the entire hippocampus is compared to the regions that it is structurally and functionally closely related to, hippocampal size is actually decreasing relative to the neocortex along the primate lineage and smallest in humans (de Winter and Oxnard, 2001)—a trend also observed in a taxonomically more diverse species sample (Reep et al., 2007). With the further expansion of cortical area, this decline is accentuated in cetaceans (whales and dolphins), in which the hippocampus appears both very small and, at least cytoarchitecturally, not well differentiated (Jacobs et al., 1979; Morgane and Jacobs, 1986; Patzke et al., 2015).

The discrepant views on the size of CA1 reflect two mechanistic models of changes in the size of brain regions—predictable, rule-based changes (linked regularities, Finlay and Darlington, 1995) generated by developmental and functional constraints (Finlay and Darlington, 1995; Whiting and Barton, 2003; Herculano-Houzel, 2011; Charvet et al., 2015) and mosaic changes (Harvey and Krebs, 1990; Barton and Harvey, 2000; de Winter and Oxnard, 2001; Rehkämper et al., 2008), which reflect deviations from the size that rules would predict. Linked regularities can explain size relationships across orders of magnitude and quantitatively dominate the variability in the size of brain components across species. Although smaller in size, mosaic changes can reflect both taxonomic relations and life-style groups (de Winter and Oxnard, 2001; Oxnard, 2004), and anatomically highly localized changes have been associated with speciation events (terminal fields of hippocampal afferents, Slomianka and West, 1989) or specific behavioral adaptions (lateral geniculate parvo-/magnocellular cell ratio, Finlay et al., 2014). In a recent study (Slomianka et al., 2013), we observed differences in the relative sizes between hippocampal principal cell populations that in part were shared by a number of taxonomically related species and in part appeared to be species specializations. Three rodent species fell within a tight group that was characterized by a relatively large CA3 cell population. Instead, primates had relatively large hilar and CA1 cell populations, with further emphasis on CA1 but less emphasis on the hilus in humans. One aim of this study was to test if these observations are robust to the inclusion of additional species and to define the cell populations that may quantitatively differentiate species within the rodent group. To this end, we expanded the number of species/strains available for analysis to 20, by generating hippocampal principal cell number estimates for eleven additional rodent species of different taxonomic groups and occupying distinctly different habitats. We also include estimates obtained from one additional primate species.

We previously analyzed some of these species for adult hippocampal neurogenesis (AHN; reviewed in Amrein et al., 2011; Amrein, 2015). While differences in AHN could be related to natural habitat differences (e.g., Cavegn et al., 2013) or selective pressures exerted by humans (Huang et al., 2015), it is not clear how differences in AHN are related to other changes in the network that they are part of. This applies to both the identity and direction of change in other populations as well as to the relative size of changes in the small cell populations that represent AHN as compared to those in other, much larger cell populations. To begin answering these questions, we generated estimates of proliferating cell numbers and young neurons for four species and we extended previous estimates of two additional species. This allowed the joint analysis of AHN-related and principal cell numbers in nine rodent species.

## Materials and methods

### Animals

**Figure 1:**
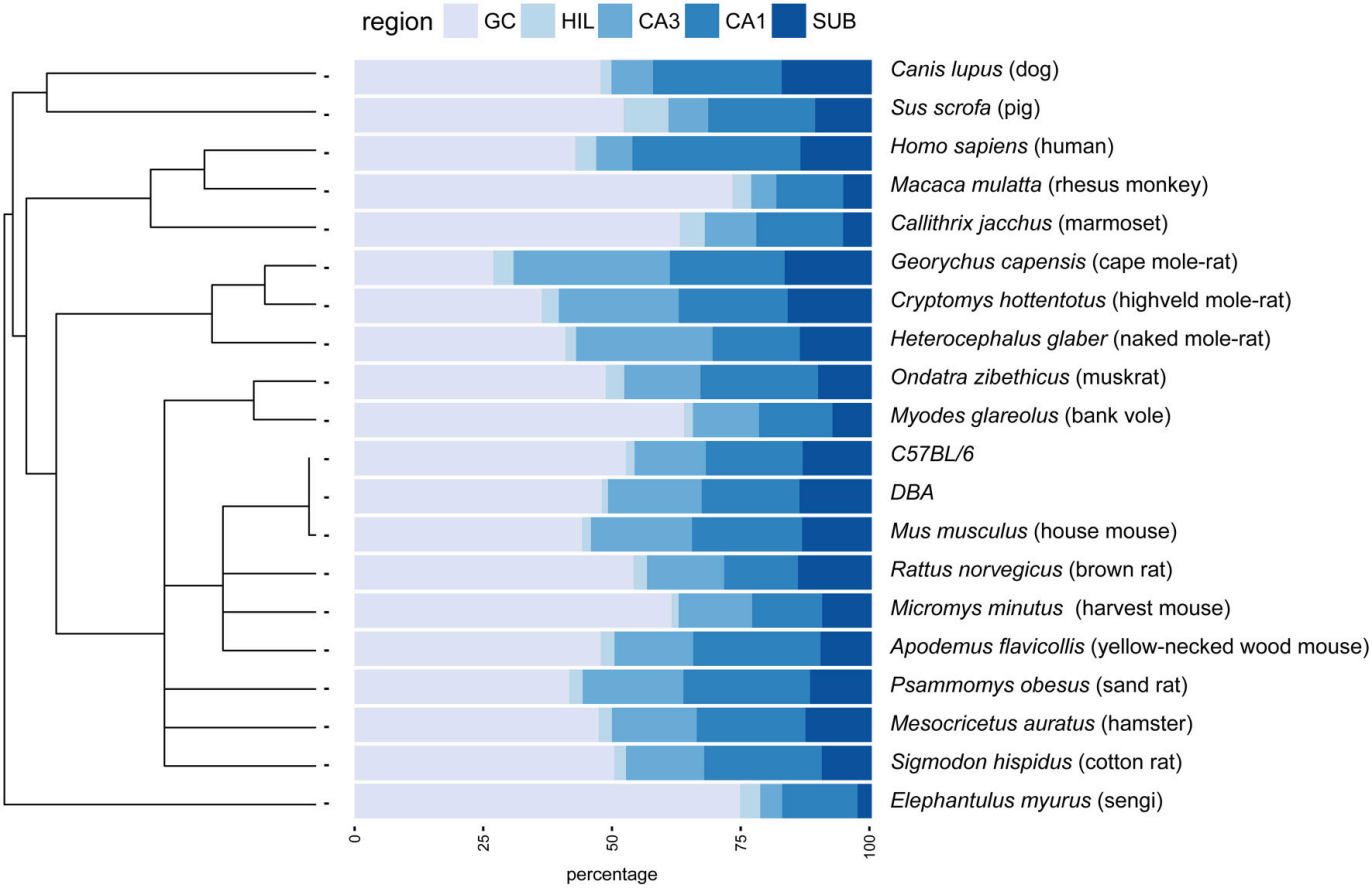
**Principal hippocampal cell number distribution in the phylogenetic tree**. A rooted phylogenetic tree (Fritz et al., 2009) of the 20 species and strains used in the study is shown along the relative size of the hippocampal cell populations in percentages. Species with extreme relative values for granule cells (GC) disperse over the tree (rhesus monkey, marmoset, bank voles, sengi), while relative high CA3 values are prevalent in rodents. GC, granule cells; HIL, hilus; SUB, subiculum.

A total of 18 unique species, with one of the species represented by three strains, were analyzed in the study. Estimates of hippocampal principal cell numbers of eleven species (cotton rat, hamster, sand rat, bank vole, house mouse: wild-type, C57BL/6 and DBA, muskrat, yellow necked wood mouse, naked mole-rat, highveld mole-rat, cape mole-rat, and marmoset) were performed for this study. Harvesting of brain tissue was performed in agreement with Canton of Zurich veterinary office guidelines. Principal cell number estimates for seven further species were taken from previously published results [sengi, house mouse (C57BL/6), harvest mouse, rhesus monkey, human, brown rats (Wistar and Sprague-Dawley), pigs and dogs]. Proliferating (Ki67+) cell and young (DCX+) neuron numbers were estimated in four species for this study (hamster, sand rat, cotton rat, muskrat). Existing proliferating cell numbers of yellow-necked wood mouse and bank vole were complemented with DCX+ neuron number estimates. Neurogenesis data for the remaining rodent species were taken from previous publications. Table 1 provides a full overview of the species and data sources. The data for the house mouse was analyzed by way of three groups: C57BL/6, DBA and wild-type house mouse, resulting in a total of 20 analyzed groups of species or strains. Sprague-Dawley and Wistar rats were pooled because wild-type estimates are not available. Phylogenetic relations between the species used are illustrated in Figure 1.

**Table 1:** **Overview of the species**.

**Species**	**Sex:N**	**Mean age in month (SD)**	**Further information**
House mouse, wild-type^*^ (*Mus musculus domesticus*)	m:5	3.5	Rodentia, Muridae F1 from wild-caught; (Klaus et al., 2012)
House mouse, DBA^*^	f:6	3	Rodentia, Muridae; (van Dijk et al., in press)
House mouse, C57BL/6	f:11; m:2	3	Rodentia, Muridae; (van Dijk et al., in press) and (Fabricius et al., 2008)
Rat, Sprague-Dawley (*Rattus norvegicus*)	m:5	~5	Rodentia, Muridae; (Fitting et al., 2010)
Rat, Wistar	m:5; f:5	1.5 (0.5)	Rodentia, Muridae; (West et al., 1991; Hosseini-Sharifabad and Nyengaard, 2007)
Yellow-necked wood mouse^*^^&^ (*Apodemus flavicollis*)	f:2; m:4	4.3 (0.5)	Rodentia, Muridae; (Amrein et al., 2004a)
Harvest mouse (*Micromys minutus*)	n/a:5	n/a, adult	Rodentia, Muridae; (Slomianka et al., 2013)
Sand rat^*^^&^ (*Psammomys obesus*)	m:6	2.7 (0.3)	Rodentia, Cricetidae Harlan Laboratories, Israel
Bank vole^*^^&^ (*Myodes glareolus*)	f:1; m:3	7.4 (6.9)	Rodentia, Cricetidae; (Amrein et al., 2004a)
Muskrat^*^^&^ (*Ondatra zibethicus*)	f:3; m:3	9.5 (4.2)	Rodentia, Cricetidae wild-caught, Germany
Hamster^*^^&^ (*Mesocricetus auratus*)	m:6	2.6 (0.2)	Rodentia, Cricetidae Harlan Laboratories, Netherlands
Cotton rat^*^^&^ (*Sigmodon hispidus*)	m:6	2 (0.1)	Rodentia, Cricetidae Harlan Laboratories, Netherlands
Highveld mole-rat^*^ (*Cryptomys hottentotus*)	f:6	20.3 (9.2)	Rodentia, Bathyergidae; (Amrein et al., 2014)
Cape mole-rat^*^ (*Georychus capensis*)	f:4; m2	26 (10.7)	Rodentia, Bathyergidae; (Amrein et al., 2014)
Naked mole-rat^*^ (*Heterocephalus glaber*)	f:1; m:4	39.3 (2.8)	
Eastern rock sengi (*Elephantulus myurus*)	f:4; m:4	8.8 (1.8)	Macroscelidea, Macroscelididae; (Slomianka et al., 2013)
Dog (*Canis lupus familiaris*)	n/a:10	109.1 (63.3)	Carnivora, Canidae; (Siwak-Tapp et al., 2008)
Pig, domestic (*Sus scrofa domestica*)	f:5	3.2	Artiodactyla, Suidae; (Holm and West, 1994)
Common marmoset^*^ (*Callithrix jacchus*)	f:2; m:3	53.6 (41.6)	Primates, Callitrichidae; (Amrein et al., 2015)
Rhesus monkey (*Macaca mulatta*)	m:8	14 (21.4)	Primates, Cercopithecidae; (Keuker et al., 2003)
Human (*Homo sapiens*)	f:17; m:56	777 (260.8)	Primates, Hominidae; (West and Gundersen, 1990; West, 1993; Simic et al., 1997; Harding et al., 1998; Korbo et al., 2003)

Superscripts mark groups for which principal cell number estimates (asterisk) and neurogenesis (ampersand) data are presented for the first time in this study.

### Histology and immunohistochemistry

To estimate principal cell population sizes, the left hemisphere of each animal was fixed in 4% paraformaldehyde, dehydrated and embedded in glycol methacrylate (Technovit 7100, Heraeus Kulzer GmbH, Wehrheim/Ts, Germany) according to the manufacturer's instructions but with extended infiltration times depending on brain size (Amrein and Slomianka, 2010). Series of every sixth 20 μm sections were mounted, dried and stained in Giemsa solution (1.09204.0500, Merck) diluted 1:10 in 67 mmol KH_2_PO_4_ buffer (Iñiguez et al., 1985). The stained sections were differentiated 10 s in 1% acetic acid, dehydrated and coverslipped.

Immunohistochemistry for the neurogenesis markers Ki67 (proliferation) and DCX (neuronal differentiation) was performed as described in detail in previous reports on neurogenesis in these species (Table 1). Briefly, 40 μm thick sections were cut from the frozen right hemispheres. Complete series of free-floating sections were washed with Tris-Triton (TBS pH 7.4 with 0.05% Triton). Antigen retrieval was performed using 10% citric acid (DAKO) at 90°C for 45 min (Ki67) or by short microwaving (DCX, 2 min). After washes in Tris-Triton, sections were incubated in 0.6% H_2_O_2_ in TBS with 0.1% Triton to block endogenous peroxidase. After further washes, the sections were incubated in appropriate blocking solution (2% serum, 0.2% Triton in TBS) for 1 h. Primary antibody concentration was titrated for each species to generate near saturation of the signal at low background levels. Primary antibodies (Ki67: polyclonal rabbit-anti-Ncl Ki67, Novocastra or Mouse anti-Ki67, BD Pharm and DCX: polyclonal goat-anti-doublecortin, Santa Cruz) were diluted with blocking solution and applied overnight at 4°C. With intermittent washes in TBS, sections were immersed in biotinylated secondary antibodies diluted in TBS with 2% serum, ABC solution (PK-6100, Vector Laboratories) and diaminobenzidine (D4418-50SET, Sigma) following the manufacturers' instructions. All sections were then dehydrated and mounted.

### Cell number estimation

Hippocampal principal cell numbers were estimated using the optical Fractionator (West et al., 1991) with StereoInvestigator 10 software (MBF Bioscience, Williston, VT, USA). Every 12th, 18th, or 24th section (every 2nd, 3rd, or 4th section of the series) was sampled with 10 μm high dissectors and 2 μm top guard zones. Section thickness was estimated at every 10th sampling site. Sampling parameters, cells counts, total number estimates based on number-weighted section thickness (Dorph-Petersen et al., 2001), coefficients of error (CEs) of the individual estimates for *m* = 0 (Gundersen et al., 1999; Slomianka and West, 2005) and CE^2^/CV^2^ ratios are listed in Table 2.

**Table 2:** **Estimates of unilateral hippocampal cell numbers and sampling parameters**.

	**DBA**	**C57BL/6**	**House mouse**	**Highveld mole-rat**	**Cape mole-rat**	**Naked mole-rat**	**Sand rat**	**Hamster**	**Cotton rat**	**Muskrat**	**Yellow-necked wood mouse**	**Bank vole**	**Marmoset**
**Granule cells**	409,981	438,187	476,984	588,647	48,8676	380,255	611,424	497,717	613,446	1415,956	1437,135	204,2739	249,8487
SD	22,614	137,143	72,686	93,514	77,599	34,806	112,574	88,880	75,937	82,545	213,879	211,439	431,224
Mean CE	0.13	0.13	0.09	0.08	0.11	0.07	0.10	0.09	0.08	0.10	0.07	0.07	0.07
CE^2^/CV^2^	5.45	1.06	0.34	0.32	0.45	0.70	0.30	0.27	0.42	2.76	0.24	0.40	0.18
Frame/grid size	7/100	7/100	10/100	12/140	18/324	10/120	12/160	12/120	12/120	45/180	10/210	10/210	15/120
Sections	12 (0.8)	11(1.6)	20 (1.1)	15 (0.2)	16 (0)	21 (0.4)	16 (1.2)	15 (0.8)	16 (0.6)	16 (2.8)	31 (1.8)	25 (4.7)	13 (1.5)
Cells counted	145 (60)	144 (16)	204 (99)	216 (30)	164 (62)	353 (23)	210 (40)	221 (27)	240 (34)	213 (52)	263 (33)	380 (88)	697 (78)
**Hilar cells**	10,487	13,029	18,561	53,219	71,216	19,365	37,742	26,769	27,658	104,394	78,578	53,695	187,457
SD	1212	2267	3099	5697	12,930	2765	5494	5894	3033	15,103	7406	6856	12,391
Mean CE	0.13	0.13	0.11	0.13	0.13	0.15	0.08	0.10	0.08	0.16	0.09	0.13	0.104
CE^2^/CV^2^	1.37	0.49	0.47	1.26	0.60	1.17	0.33	0.20	0.58	1.32	0.82	1.01	2.52
Frame/grid size	30/70	30/70	30/70	40/120	30/150	40/70	45/140	45/120	45/120	45/210	40/170	40/140	40/200
Sections	12 (1.2)	11 (1.6)	9 (0.9)	9 (0.4)	10.6 (1.2)	9 (0.4)	16 (1.0)	15 (1.7)	15 (0.8)	16 (2.3)	14 (1.7)	8 (1.0)	13 (1.3)
Cells counted	148 (48)	126 (19)	215 (36)	199 (19)	91 (21)	386 (61)	223 (22)	163 (32)	163 (11)	171 (36)	220 (38)	168 (15)	159 (19)
**CA3 pyramids**	155,129	113,603	21,3077	374,376	55,0413	24,9021	285,537	171,799	184,583	432,250	454,510	409,606	392,291
SD	16,621	29,322	36,953	28,207	71,336	44,854	56,815	27,378	21,957	61,979	47,889	77,249	35,139
Mean CE	0.12	0.11	0.12	0.1	0.1	0.08	0.10	0.10	0.08	0.11	0.09	0.09	0.09
CE^2^/CV^2^	1.23	3.71	0.51	1.61	0.66	0.18	0.27	0.36	0.47	0.61	0.65	0.26	0.95
Frame/grid size	11/100	11/100	11/100	18/160	15/200	15/130	25/250	25/160	25/160	25/260	15/190	15/140	30/200
Sections	15 (1.2)	13 (1.7)	10 (0.8)	11 (0.4)	12.5 (1.2)	12 (0.4)	18 (1.5)	18 (1.0)	18 (0.8)	20 (2.9)	14 (1.5)	9 (0.5)	13 (1.1)
Cells counted	146 (47)	97 (10)	165 (20)	153 (8)	101 (17)	205 (17)	184 (39)	179 (25)	187 (15)	138 (38)	142 (14)	185 (20)	185 (29)
**CA1 pyramids**	162,744	155,745	233,670	338,455	415,012	158,693	359,291	221,382	275,219	663,434	740,667	455,249	656,509
SD	21,729	51,368	56,389	29,375	132,131	18,884	70,170	34,246	11,044	81,379	91,568	93,897	41,394
Mean CE	0.13	0.14	0.10	0.12	0.12	0.10	0.11	0.10	0.08	0.10	0.10	0.10	0.12
CE^2^/CV^2^	1.09	1.51	0.17	2.12	0.15	0.76	0.32	0.44	3.51	0.65	0.60	0.24	4.54
Frame/grid size	11/100	11/100	11/100	18/160	15/180	15/110	25/250	25/160	25/160	25/260	10/160	15/130	30/320
Sections	16 (2.8)	13 (1.7)	12 (0.6)	12 (0.8)	14 (1.4)	13 (0.7)	19 (1.2)	18 (1)	18 (0.8)	20 (2.9)	16 (1.6)	10(0.5)	14 (1)
Cells counted	153 (66)	130 (8)	174 (23)	141 (12)	97 (25)	183 (11)	236 (7)	230 (29)	280 (18)	216 (63)	154 (14)	230 (37)	173 (54)
**Subicular cells**	119,944	108,479	14,7146	263,138	30,911	13,1505	173,602	134,379	117,120	302,230	295,464	241,765	216,992
SD	12,015	22,932	32,839	33,554	62,378	33,019	28,260	18,230	9493	31,484	38,801	42,119	31,991
Mean CE	0.11	0.10	0.1	0.09	0.10	0.11	0.08	0.07	0.08	0.09	0.09	0.10	0.12
CE^2^/CV^2^	1.16	0.57	0.21	0.50	0.26	0.13	0.25	0.28	0.95	0.72	0.42	0.30	0.67
Frame/grid size	25/140	18/140	18/140	20/150	20/190	20/130	45/320	45/200	45/190	45/350	20/180	20/140	18/240
Sections	15 (1.4)	12 (1.7)	12 (0.9)	12 (0.5)	13 (1.6)	12 (1.1)	18 (1.2)	18 (0.8)	18 (0.8)	18 (3.4)	15 (1.5)	11 (0.6)	14 (1.7)
Cells counted	156 (59)	128 (13)	145 (24)	154 (20)	112 (22)	192 (35)	259 (100)	286 (32)	282 (20)	179 (23)	176 (23)	183 (20)	132 (60)

Principle cell numbers of animals analyzed for this study. Numbers presented here are means. Frame and grid sizes are in μm. Sections and cells counted are presented as mean (SD).

Ki67- and DCX-positive cells were quantified under a x63 oil-immersion lens and either counted manually and exhaustively (Ki67) but avoiding cells in the top focal plane of the section or by using the optical Fractionator (DCX). For details, see Table 3 and publications in Table 1.

**Table 3:** **Neurogenesis related cell counts**.

	**DBA**	**C57BL/6**	**House mouse**	**Hamster**	**Sand rat**	**Cotton rat**	**Muskrat**	**Yellow-necked wood mouse**	**Bank vole**
**Proliferating cells**	1753	4505	1630	2813	2452	5053	2487	15,030	5373
SD	184	765	268	1240	591	2378	657	3556	2830
Mean CE	0.07	0.06	0.07	0.07	0.10	0.09	0.09	0.06	0.08
CE^2^/CV^2^	0.42	0.11	0.19	0.03	0.17	0.04	0.11	0.06	0.02
Frame/grid size	Exhaustive counts								
Sections	14 (0.8)	14 (2.1)	13 (1.2)	7 (0.5)	8 (1.3)	8 (1.2)	10 (1.2)	12 (3)	14 (1)
Cells counted	351 (37)	901 (153)	326 (53)	281 (124)	245 (59)	505 (18)	178 (47)	2505 (593)	896 (472)
**Young neurons**	7372	21,080	10,677	7450	10,850	9103	11,655	57,682	38,833
SD	940	3072	2609	1069	3587	1484	3781	4382	24,082
Mean CE (*m* = 0)	0.11	0.07	0.09	0.07	0.12	0.05	0.08	0.10	0.08
CE^2^/CV^2^	0.83	0.25	0.14	0.26	0.15	0.10	0.06	1.59	0.02
Frame/grid size	40/100	25/100	30/125	35/60	Exhaustive counts			20/100	30/100
Sections	15 (1.1)	14 (1.0)	13(1.6)	8 (0.8)	9 (1.0)	9 (0.8)	9 (1.2)	7 (0.8)	7 (0)
Cells counted	200 (42)	315 (96)	123 (30)	267 (51)	1085 (359)	902 (155)	817 (255)	210 (47)	376 (93)

Estimated numbers of proliferating cells and young neurons in the hippocampus of rodents. Numbers are given unilateral and represent means; no correction for age was made. Proliferating cell numbers for yellow-necked wood mice and bank voles published earlier (Amrein et al., 2004b) were included for convenience. Frame and grid sizes are in μm. The number of sections used and cells counted to generate estimates of total number are presented as mean (SD).

### Age normalization of neurogenesis related cell counts

**Figure 2:**
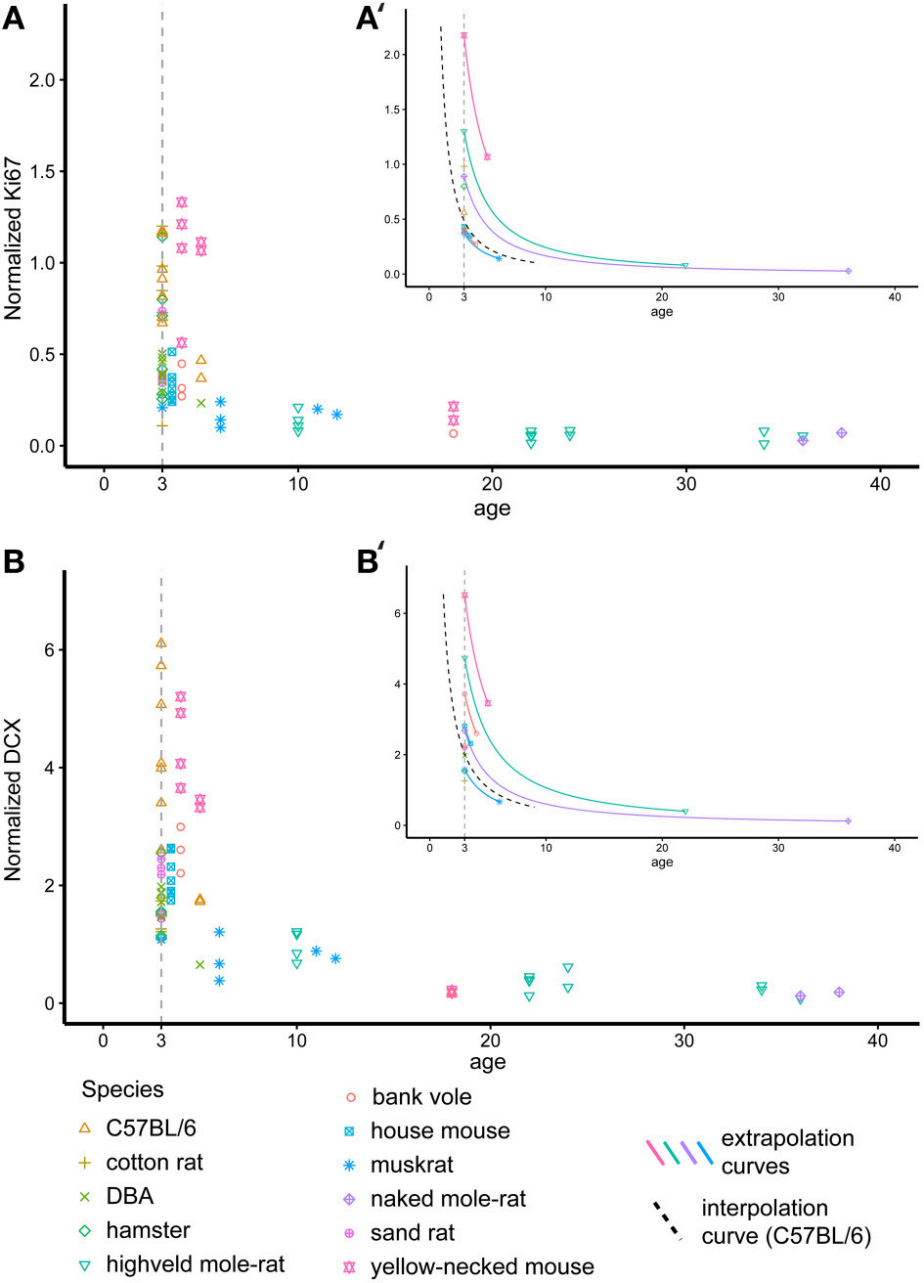
**Recalculating neurogenesis in rodents to a common age**. Hippocampal cell proliferation (Ki67, **A**) and young neurons (DCX, **B**) differ extensively due to large age differences between animals. For further analysis, cell numbers were therefore extrapolated for each individual animal to a common age of 3 months based on the negative exponential curve seen in laboratory C57BL/6 (Ben Abdallah et al., 2010, dashed black line in **A**′**,B**′). **(A**′,**B**′**)** exemplarily visualizes the procedure on single animals. For graphical presentation, neurogenesis-related cell numbers are given as percentage of total granule cell number (Normalized Ki67 and DCX, respectively).

The ages (known or estimated) of the rodents in this study varies between 1 and 42 months (Table 1, Figure 2). In order to compare neurogenesis between animals, we aimed to recalculate neurogenesis-related cell counts to a common age. We chose 3 months, as the majority of animals in our data sample were close to this age. The decline of neurogenesis with age is independent of life history or the expected life span of the species and can best be described using a negative exponential model (Amrein et al., 2011). The exponential curve of both Ki67+ and DCX+ cells in C57BL/6 over 9 months was reported by Ben Abdallah et al. (2010). This known exponentional model was used to virtually move older or younger animals along this curve to the common age of 3 months (Figures 2A′,B′) according the following equation

$$\begin{array}{l} Cell\;Number_{3-month\;age\;estimate}=\\ Cell\;Number_{actual\;estimate}\;\times \;e^{\left[\ln\left(3\right)-\ln\left(actual\;age\right)\right]\times Y} \end{array}$$

For Ki67 and DCX estimates of *Y* were reported to be −1.3933 and −1.2407, respectively (Ben Abdallah et al., 2010). In addition, the decline of both DCX+ and Ki67+ cell numbers with age was also estimated based on all rodents in this study. The ages of wild-caught animals were estimated by the time of capture and breeding time, lens weight (Barker et al., 2005) and bone lines (Cavegn et al., 2013). In this recalculation, *Y* was estimated to be −1.1929 for Ki67 and −1.0798 for DCX.

### Statistics

Large differences exist between the principal cell populations within species (e.g., in bank voles granule cells are more than 30 times more numerous than hilar cells) and between species (see Table 2). To account for these large differences, values, unless otherwise stated, were log transformed and scaled by subtracting the mean of all neuron populations of each individual animal from the individual population estimates and dividing the result by the standard deviation of the mean. After this transformation, all animals therefore have cell counts with a mean of zero and a standard deviation of one across cell populations, but the relative size differences between populations within each animal are retained.

A brief numerical example may make the data transformation more accessible. In one specific animal XY, we estimate there to be 90 granule cells, 10 hilar cells, 30 CA3 cells, 40 CA1 cells and 30 subicular cells. The mean of the population estimates in this animal is 40 with a standard deviation (SD) of 30. The mean is subtracted from individual estimates, which results in values of 50 (granule cells), −30 (hilar cells), −10 (CA3 cells), 0 (CA1 cells), and −10 (subicular cells). The new mean of the population is 0. After dividing by the SD, the values are 1.66, −1, −0.33, 0, −0.33. The mean is still 0, but the SD of the data is now 1. Note that absolute differences are preserved after the subtraction of the mean. Also, relative differences still are preserved after the division by the SD. Applying this transformation to all individuals of all species provides directly comparable values that are independent from differences in absolute size and associated differences in variance.

For the analysis of the connectivity of cell populations (convergence or divergence between the functionally connected neurons of granule cells to hilus cells, granule cells to CA3 cells, CA3 cells to CA1 cells, and CA1 cells to subicular cells), ratios were used, requiring no further transformation.

The relationship between species and hippocampal cell population sizes was investigated using a correspondence analysis which is a statistical method for visualizing the associations (degree of correspondence) between the levels of a multi-way contingency table (Greenacre and Hastie, 1987). Correspondence analyses are applied using the R package “MADE4” (Culhane et al., 2005). It calculates the chi-squared distances between the actual and expected values for both columns (cell numbers in hippocampal regions) and rows (species). Chi-square statistics can be used to examine small tables; correspondence analysis allows for the simplification of large tables with many columns and/or rows (Greenacre and Hastie, 1987). This analyses allows complex data to be reduced to a two-dimensional plot while still capturing the majority of the variance in our data (>80%). In all plots presented, the x-axis represents the first dimension of the correspondence analysis while the y-axis represents the second dimension.

## Results

### Hippocampal morphology

**Figure 3:**
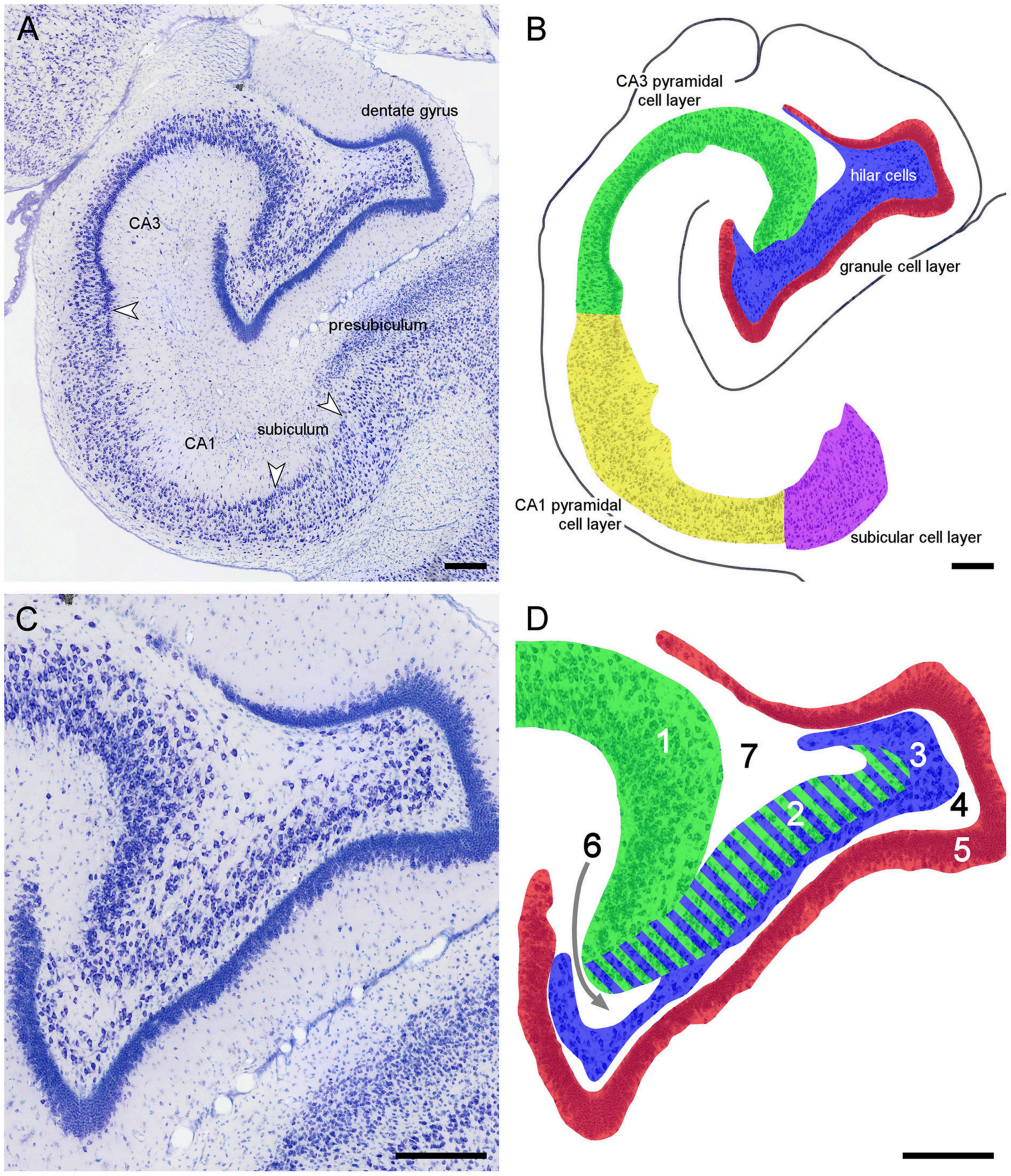
**Principal hippocampal subdivisions in the marmoset**. **(A)** Major hippocampal subdivisions in Giemsa-stained marmoset mid-septotemporal hippocampus. Arrows mark the boundaries between subdivisions at the level of the cell layer(s). **(B)** Definitions of the principal cell population in the marmoset mid-septotemporal hippocampus. **(C)** Complex hilar cytoarchitecture of the marmoset dentate gyrus that is common in non-rodent species. **(D)** Definitions of the regions that have been used to define CA3 and hilar cell populations within the dentate gyrus; **1**: CA3 or CA3o (outer CA3, Houser et al., 1990), **2**: reflected blade of CA3 (Lorente De Nó, 1934) or CA3h (used in this study; Lim et al., 1997; Ding and Van Hoesen, 2015) or CA3i (inner CA3, Houser et al., 1990) or CA4 (Rosene and Van Hoesen, 1987), **3**: polymorphic cell layer (**2**+**3**: CA4 of Lorente De Nó, 1934), and **4**: plexiform layer (Cajal, 1968), **5**: dentate granule cells. Stratum radiatum (**6**) and stratum oriens (**7**) insert themselves superficial and deep to CA3h. The separation between CA3h and the hilar polymorphic layer is variable in different species and at different septotemporal levels. When the CA3h and the polymorphic cell layer merge, we cannot reliably distinguish CA3h cells from hilar polymorphic cells in Nissl-stained preparations. Scale bars: 250 μm.

**Figure 4:**
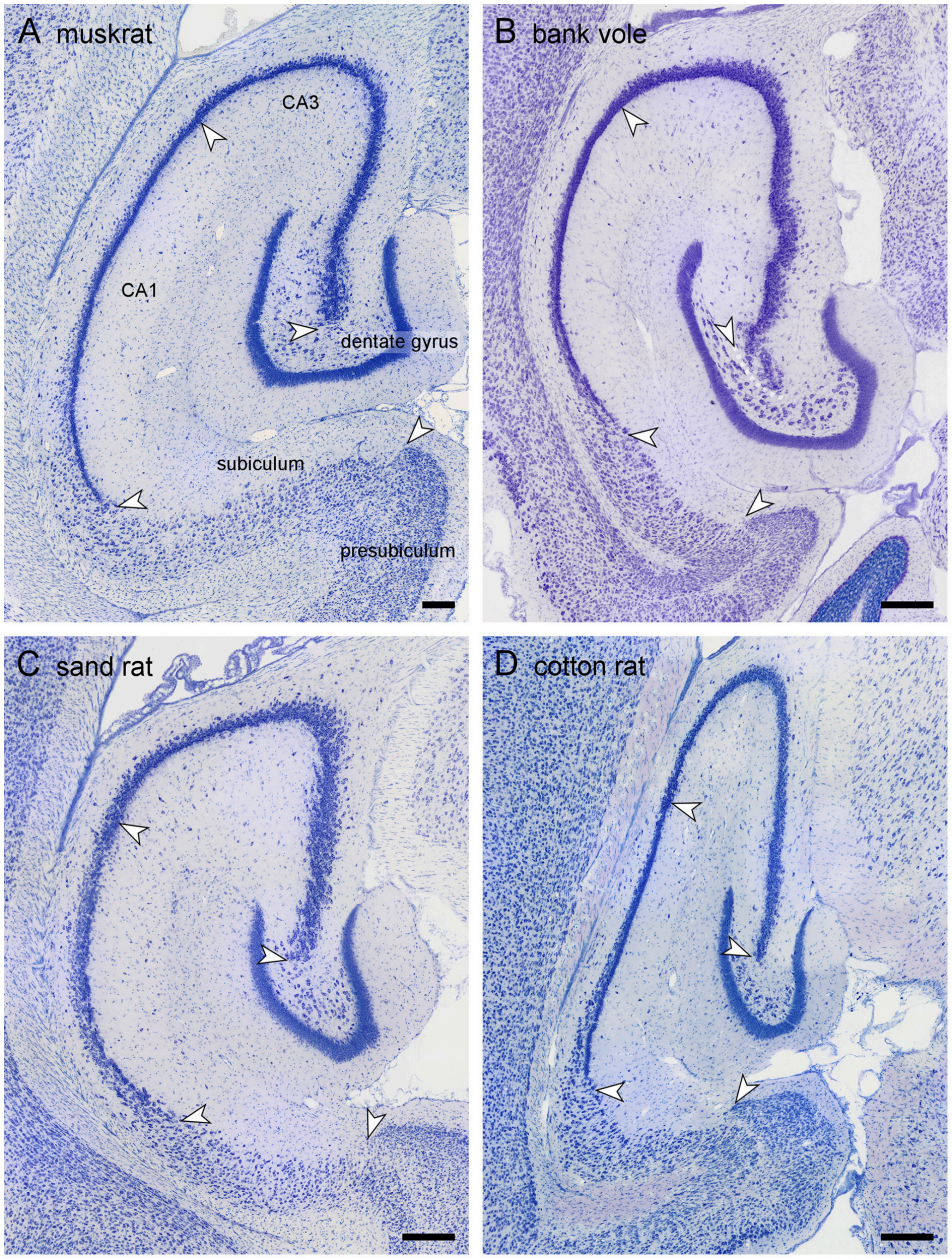
**Hippocampal morphology of the four rodent species**. Mid-septotemporal hippocampus of the four rodent species presented for the first time: **(A)** muskrat, **(B)** bank vole, **(C)** sand rat, and **(D)** cotton rat. The brains were sectioned horizontally, and images were taken in sections immediately following the disappearance of the septal pole of the dentate gyrus. Arrows mark the boundaries between subdivisions at the level of the cell layer(s). Scale bars: 250 μm.

Because of the variable nomenclature of CA3 pyramidal cells close (proximal) to the dentate gyrus and recent revisions, this region and the borders that it contains is illustrated in Figure 3 for the marmoset monkey. Cytoarchitectural differentiation in marmoset monkeys largely corresponds to that seen in all non-rodent species included in this study. Hippocampal cytoarchitecture and the boundaries between hippocampal fields have not previously been illustrated for the muskrat, cotton rat, sand rat and bank vole and are shown in Figure 4. Cytoarchitecture and septotemporal changes in these species follows the general pattern described for the rat and mouse (e.g., Haug, 1974; West et al., 1978).

Similar to the house mouse, in three of the species (muskrat, sand rat and bank vole) a well-defined cell-poor dentate plexiform layer is located between the granule cell layer and the hilar polymorphic cells. It is not consistently present in cotton rats. Similar to all other rodents included in this study, none of the species show a reflected blade of CA3. In all species, CA1 pyramidal cells are markedly smaller than CA3 pyramids, and the change in cell size was used to define the CA1/CA3 border. A separate CA2 could not be defined reliably in any of the four species based on cytoarchitectural criteria. The CA1 pyramidal cell layer is distinctly divided into deep and superficial sublayers in sand rats and bank voles throughout most of the proximodistal and septotemporal extent of CA1. While the layer appears very compact in bank voles, cells are quite loosely packed in sand rats. More similar to the house mouse and rat, lamination within CA1 develops gradually from septal-distal to proximal-temporal in muskrats and cotton rats. Proximal and distal divisions of the subiculum are better defined in muskrat and cotton rat than in the bank vole or sand rat. Differences in the size of hippocampal fields are readily apparent upon visual examination and reflect the sizes of the principal cell population that are described below.

### Principal hippocampal cell numbers

The means of total cell number estimates per hippocampal region are listed in Table 2. Using a conservative smoothness constant (m) of 0 (Slomianka and West, 2005), the overall mean CE of the estimates was ~0.10. CE^2^/CV^2^ were typically below 0.5, indicating that variability contributed by the estimation procedure was a minor source of the total variability of cell number estimates within each species (Table 2). Species in which the CE^2^/CV^2^ were higher than 0.5 were investigated and all cases could be attributed to unusually small variation between animals. Several species in the study were composed of animals of both sexes, but low per-sex *n* prevented statistical testing for possible sex effects.

**Figure 5:**
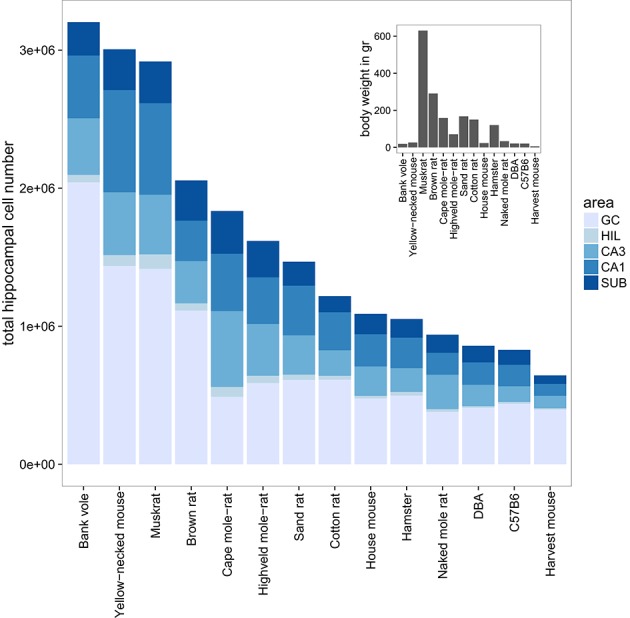
**Hippocampal cell number and body weight relationships in the rodent group**. Total cell numbers were estimated for each of the five main hippocampal region: granule cells (GC), hilus (HIL), CA3, CA1, and the subiculum (SUB). The mean number of estimated cells per region and species are plotted in a bar plot. The 14 rodent species and strains are sorted according to total hippocampal cell number. The insert shows the corresponding mean bodyweight for each of the 14 species and strains.

The largest hippocampi with regard to both total cell number and volume are, not surprisingly, found in the largest species in our data set: humans, monkeys, dogs and domesticated pigs. Notable species are the Eastern rock sengi (Slomianka et al., 2013), yellow-necked wood mouse and bank vole that are all small animals (< 50 g) with a number of hippocampal cells comparable to much larger species such as dogs, muskrats, and marmosets (Figure 5 for rodent comparison). The relative sizes of the cell population are roughly similar between species (see Figure 1). Granule cells in the dentate gyrus form the largest population in all species, the second largest cell population is that of CA1 followed by CA3, the subiculum and last, with the smallest cell population, the hilus. There are however species showing exceptions to this pattern. The three mole-rat species all have CA3 cell numbers that exceed the number of CA1 cells (Figures 1, 5). Second exceptions are the four largest species in our data set (human, rhesus monkey, pig and dog, see Figure 1), in which subicular cell numbers exceed those in CA3.

### Correspondence analyses of all species

**Figure 6:**
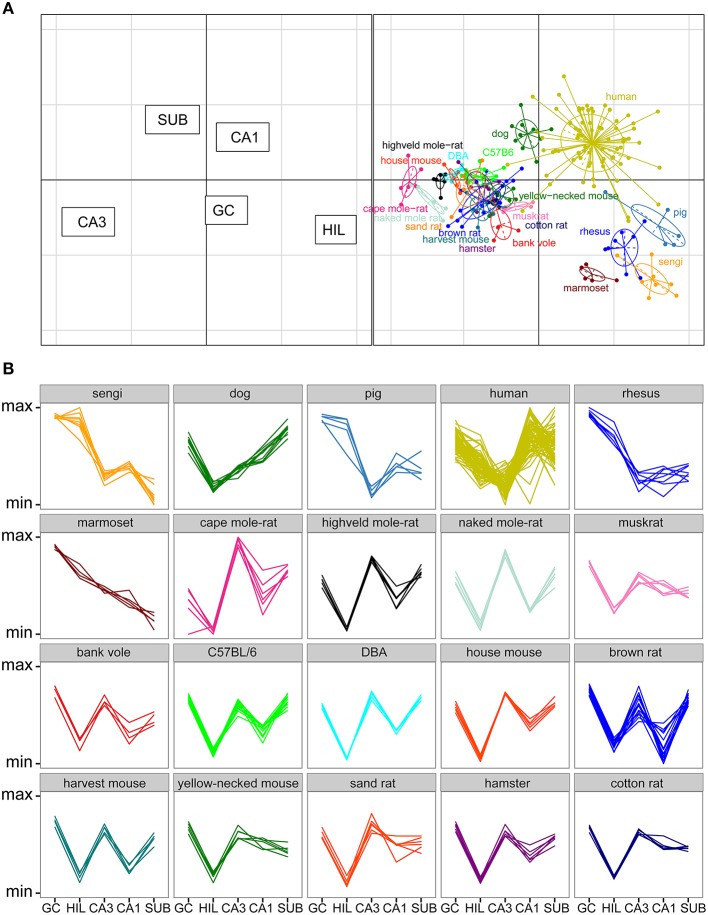
**Species clusters occupy distinct spaces in the correspondence analysis**. **(A)** Correspondence analysis showing the relationships between species and hippocampal principal neuron numbers. Species form distinct clusters with taxonomically related species such as the rodents clustering close together. The spatial arrangement of hippocampal fields (left graph) can be used to determine which populations are driving the species clustering. Rodents, especially mole-rats, have relatively high numbers of cells in the CA3 and relatively few cells in the CA3h/hilus (right graph). HIL, CA3h/hilar cells; SUB, subicular cells; GC, dentate granule cells. **(B)** Species profile plots showing group-specific patterns in the relative composition of hippocampal principal cell populations. The y-axes range from the minimum to the maximum value for each hippocampal field across all species. For example, rodents have relatively larger CA3 than all the other species and humans have relatively larger CA1 than all other species. Each line indicates one individual animal.

Total cell numbers for each of the five principle cell populations for all 20 species or strains were compared in a correspondence analysis (Figure 6A). Data in this analysis was log transformed and scaled against the five populations per individual animal (mean of 0, standard deviation of 1), resulting in a dimensionless unit representing the relative contribution of each population to total hippocampal cell number. The two axes in Figure 6A represent 89% of the variance in the data (axis 1: 64% and axis 2: 25%). Each dot in the plot represents one animal. Hilar and CA3 populations cause the largest separation between species (respectively 38 and 35% of the variation along the first and 21 and 18% along the second axis), and they are able to separate the species into clusters, which largely align with the taxonomic grouping of the species (Figure 6A). A proportionally large CA3 in rodents results in a complete separation of all representatives of the order Rodentia from other species along the first axis. The relatively large hilar population found in the Eastern rock sengi, marmosets, rhesus monkey and pigs causes further separation. These four species, together with humans and dogs, also share a reflected blade of the CA3 pyramidal cell layer. In our study, as well as in all sources used in this study, these cells are included in the cell counts of the hilus (see Figure 3 and Discussion). Along the second axis, in addition to hilar and CA3 populations, further distinction is made by the subiculum and CA1 (26% and 19%, respectively), which results in a separation of the human hippocampus, marked by both a large CA1 and small hilar cell populations when compared to other primates. Lastly, dogs form a unique group by having both large subicular and CA1 populations. In addition to the correspondence analysis, Figure 6B shows the normalized species profiles of population sizes per animal. In these graphs the same patterns can be detected as in the correspondence analyses, where again rodent data show a distinct pattern based on the cellular composition of their hippocampi.

### Separation within the rodent cluster

**Figure 7:**
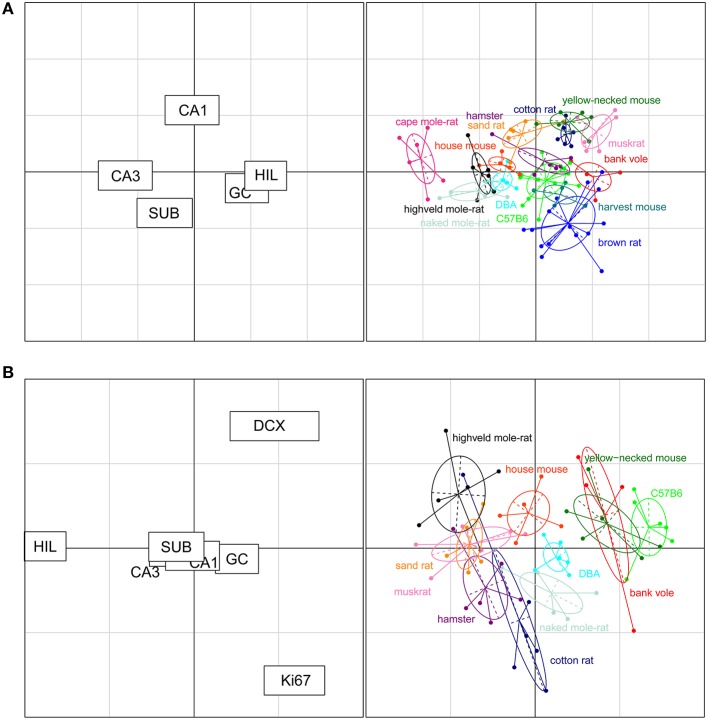
**Neurogenesis drives the separation within rodent clusters in the correspondence analysis**. **(A)** Separate correspondence analysis of the rodent cluster. Note that the range is now defined by the variability seen in rodents only, providing a higher resolution for the rodent data than in Figure 6. All cell populations contribute to the separation within the rodent cluster (left graph). For example, the three mole-rat species, house mice and DBA were pulled toward the negative direction of the x axis due to their relatively large CA3 cell population portion and small granule cell and hilus cell population portion (right graph). **(B)** When taking the neurogenic cell populations into account, the cell populations that differentiate strongly between rodents are new-born differentiating neurons (DCX+), proliferating cells (Ki67+), and hilar cells (HIL, left graph). The plot places the laboratory mouse strains C57BL/6 and DBA close to yellow-necked wood mice and bank voles. The cotton rats and hamsters (both laboratory bred) show relatively similar patterns to each other. The two mole-rat species, on the other hand, are separated from each other by their distinct levels of neurogenesis and hilar cell populations.

The rodents form a tight cluster in comparison to the other species in our data set. To further investigate the rodent group the correspondence analyses was performed using only the rodent data. Figure 7A shows this rodent-specific correspondence analysis. In doing so, the contribution of each principal cell populations is reassessed without being skewed by extreme cases seen in the other orders (e.g., the large CA1 and hilus of primates). The two axes in the plot explain 81% of the variation in the data (axis 1: 57% and 2: 24%). Within rodents, hilus and CA3 are still strong separators although their contribution is weaker than in the comparison between taxonomic orders (27% and 30% of variation along the first and 3% and 5% along the second axis). Other populations now account for more of the variation. CA1 is the main differentiator on the second axis (47%) while the granule cells significantly affect separation on both the first (25%) and second axis (11%). The entire rodent group is marked by a large CA3 (Figure 6), and CA3 variation is able to further distinguish rodents from one another. Most prominent are the three mole-rat species, which are separated from the other rodents on account of there, even for rodents, large CA3. The center of the correspondence analysis can be seen as an “average” of rodent species, this space is occupied by the two laboratory strains of the house mouse (C57BL/6 and DBA), wild-type house mouse, hamster and harvest mouse. Opposite of the mole-rat species is the brown rat, having both a small CA3 but large hilus and subiculum. Other species marked by a relatively small CA3 but average to large CA1 cell count are the bank vole, muskrat, cotton rat and the yellow-necked wood mouse.

### Adult neurogenesis as a separating factor

For 11 out of the 14 rodent groups, adult neurogenesis was assessed using the markers Ki67 for proliferating cells and doublecortin (DCX) for differentiating young neurons. The differences in age between the animals and the consequent differences in neurogenesis, was accounted for by extra-polating cell numbers estimates to an age of 3 months (Figure 2). The estimated number of proliferating cells and young neurons were taken together with the five principal hippocampal cell populations, and again analyzed in a correspondence analysis (Figure 7B).

The inclusion of the two neurogenesis parameters dramatically changes the results of the analysis due to the larger inter-species variation in neurogenesis compared to the more stable principal cell populations. The first two axes cover 83% of the variation in the data (axis 1: 60% and 2: 23%). DCX+ cells predominantly acts on the first axis (38%, second axis: 21%), and Ki67+ cells act on the second axis (48%, first axis: 2%). Surprisingly, the other hippocampal cell regions lose much of their differentiating power. The only region still having a strong effect is the hilus (first axis: 31% second: 20%). Species can be divided in four groups according to the contribution of the DCX+ cell population; bank voles, C57BL/6 and the yellow-necked wood mouse having the highest relative contribution of DCX+ cells. They are followed by the house mouse, DBA mouse and naked mole-rats having mid-high levels of DCX+ cells, subsequently followed by species with relatively low DCX+ cells: the highveld mole-rat, sand rat, muskrat, hamster and cotton rat. The separating effect of the hilar cells is interestingly enough opposite to that of the DCX+ cell population, species marked by high DCX+ cell numbers have relatively small hilar cell populations and vice versa. The Ki67+ cell population is the main separating factor on the second axis, perpendicular on the DCX+ and hilus driven first axis, where each of the four groups described show a gradient of contribution by the Ki67+ cell population (Figure 7B).

### Convergence and divergence between connected cell populations

**Figure 8:**
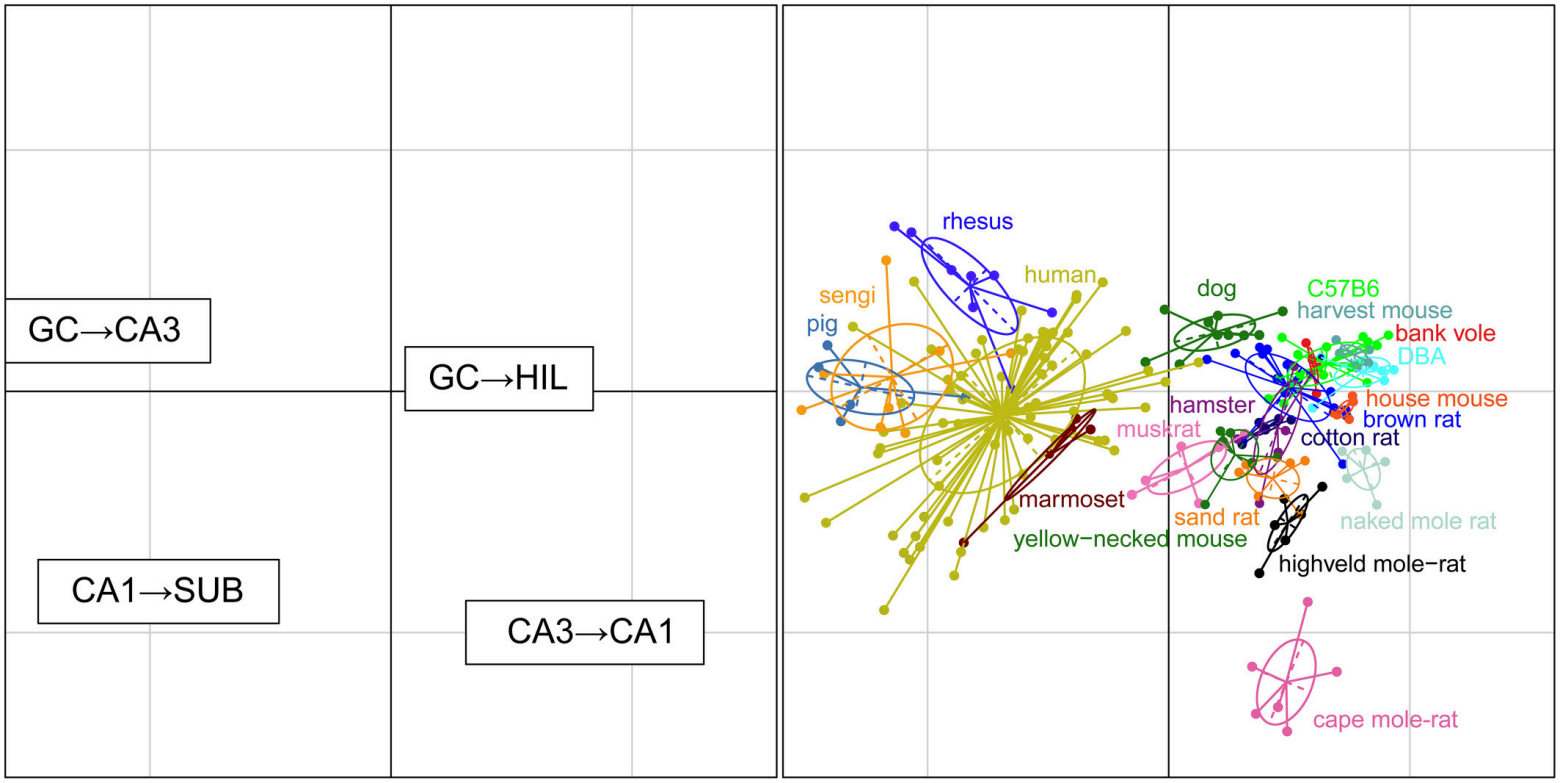
**Convergence and divergence of hippocampal cell populations across species**. While all rodents form a tight cluster similar to the figures plotting principal cell populations, all primates including humans now cluster together due to similar degrees of convergence. This indicates that individual cell populations can differ to a large degree between species, while stable convergence/divergence relationships are retained in phylogenetically related species.

For each set of interconnected cell populations, the degree of convergence/divergence was calculated as ratios (Table 4) and analyzed in a correspondence analysis (Figure 8). The values represent to what degree information may converge from many-to-few cells or diverge from few-to-many cells in the pathway. The first two axes cover 93% of the variance in the data set (axis 1: 79%, axis 2: 14%). The convergence/divergence between the GC and CA3 populations is the main differentiator with 35% of variation of the first axis, closely followed by the convergence from CA1 to subiculum and from CA3 to CA1 (CA1 to subiculum: 28% on the first and 39% on the second axis; CA3 to CA1: 24% on the first and 47% on the second axis). Rodents again form a tight cluster with the exception of the mole-rat species, marked by a much larger CA3 cell populations compared to their CA1 populations. Noteworthy is the shift between the primate species. Based on principal cell populations, humans were separated from the other primates on account of their relatively large CA1 population (Figure 6). However, the convergence/divergence values of the human data falls between the two other non-human primate species (Figure 8).

**Table 4:** **The degree of convergence and divergence of principal cell numbers in the hippocampus**.

**Species**	**GC 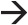 HIL**	**GC 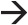 CA3**	**CA3 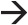 CA1**	**CA1 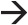 SUB**
Dog	0.041	0.165	3.044	0.694
Pig	0.166	0.144	2.686	0.513
Human	0.087	0.152	4.194	0.379
Rhesus monkey	0.048	0.061	2.599	0.421
Marmoset	0.074	0.158	1.667	0.323
Cape mole-rat	0.143	1.122	0.717	0.756
Highveld mole-rat	0.09	0.639	0.898	0.766
Naked mole-rat	0.05	0.635	0.64	0.803
Muskrat	0.072	0.302	1.526	0.452
Bank vole	0.026	0.194	1.089	0.527
C57BL/6	0.029	0.263	1.31	0.719
DBA	0.028	0.361	1.113	0.722
House mouse	0.039	0.443	1.081	0.629
Brown rat	0.047	0.231	1.013	0.993
Harvest mouse	0.022	0.228	0.942	0.697
Yellow-necked mouse	0.054	0.313	1.608	0.396
Sand rat	0.061	0.46	1.221	0.481
Hamster	0.05	0.331	1.274	0.606
Cotton rat	0.045	0.301	1.49	0.423
Sengi	0.051	0.055	3.443	0.171

Functional connectivity of the principal cell numbers in the hippocampus can be expressed as a ratio of one cell population to the next one. Convergent ratios (many cells to few cells, white in the table) apply to the granule cells to hilus cells (GC
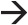
HIL), granule cells to CA3 pyramidal cells (GC
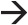
CA3), and CA1 pyramidal cells to subicular cells (CA1
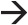
SUB). For example, C57BL/6 has a GC
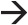
CA3 convergence ratio of 0.26 that means roughly four granule cells for each CA3 cell. Divergent ratios (few cells to many cells, gray background in the table) are found for CA3 pyramidal cells projecting to CA1 (CA3
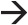
CA1). For example, C57BL/6 has a CA3
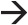
CA1 divergence ratio of 1.3, which means 1.3 CA1 neurons for each CA3 neuron. Note that in particular the mole-rat species do not follow the overall convergence—divergence pattern, see Discussion. For data sources see Table 1.

### Additional validation

Data for humans and C57BL/6 mice originated from different studies (see Table 1 for references). To test if the data sets are comparable between studies, we reanalyzed the data treating each study as a separate group. In all instances, data from different studies for a single species overlap and share the same characteristics (not illustrated). Furthermore, in addition to the primates (humans, rhesus monkeys and marmosets) in our data set, available mean values of hippocampal cell counts from an additional species (*Macaca nemestrina;* Leverenz et al., 1999) and one additional group of rhesus monkeys (Jabès et al., 2011) were included in the analysis. The data points grouped with the marmosets and rhesus monkeys respectively, supporting the outcomes of our analysis.

In view of the reported age-related changes in neuron numbers of the human hippocampus (West, 1993; Simic et al., 1997), we analyzed the combined human data set for age related differences in principal cell population numbers. Using study as a covariate, the hilar [*F*_(1, 67)_ = 6.71, *p* = 0.01], CA1 [*F*_(1, 67)_ = 22.56, *p* < 0.001] and subicular [*F*_(1, 67)_ = 15.70, *p* < 0.001] cell numbers show a significant decrease with age. We also analyzed the effect of this age related decline in cell numbers on the positioning of humans in the correspondence analyses by comparing both age-quartiles and low-age (< 30) vs. high-age (>80) groups. Both quartiles and low-age vs. high-age did show largely overlapping distributions (not illustrated).

Neurogenesis-related cell numbers in rodents of different ages where extrapolated to the age of 3 months based on two curve estimation. We compared the neurogenesis calculations (see Figure 2) based on the curve estimation of published data (Ben Abdallah et al., 2010) and in addition the curve estimation based on the data from this study (for details see in the Materials and Method Section). Using the two slopes, we found no difference in the positioning of the species relative to each other (not illustrated). While extrapolation to a common age usually did not require changing the age by more than a few months (see Figures 2A′,B′ for examples), the age of the mole-rats had to be changed by years. To investigate the impact of possible exponentially accumulating errors in extrapolated cell number estimates, the rodent sample was also analyzed excluding the mole-rats. Proliferating cells, young neurons and hilar cells remained the most powerful separating factors also when mole-rats were excluded from the species sample (not illustrated).

## Discussion

Correspondence analyses of total principal cell numbers show that the major hippocampal separating factors in either the survey of all mammalian brains included in this study or within the rodent species alone are the cell populations in the hilus and CA3. For the rodent species, neurogenesis was a key separating factor. The inclusion of new-born neurons in the analysis dramatically increased separating power of the hilar cells. Species profile plots cluster phylogenetically close species, both when comparing all species and within the rodent cluster.

### Mosaic changes in hippocampal cell composition

In rodents and primates hippocampal principal cell numbers do not decline with age (Rapp and Gallagher, 1996; Rasmussen et al., 1996; Keuker et al., 2003, 2004). Humans seem to be an exception in that the hilar, CA1 and subicular cell numbers decrease across the age range of 13–99 years. Both the large and selective effects reported previously by West (1993) or Simic et al. (1997) could be confirmed. Due to the concerted age-related changes, which largely preserved relative population sizes, there was no age-related impact on the position of humans relative to other species.

The visualization of complex data free from effects of absolute size by correspondence analyses allows comparisons of the relative compositions of the hippocampal cell populations. The analyses provided a clear separation of a tight cluster formed by rodents from other taxonomic groups and evidence for mosaic changes within the hippocampus, which agree with comparative studies at a more gross anatomical level (Barton and Harvey, 2000; de Winter and Oxnard, 2001; Reep et al., 2007). Yet, when measured against absolute cell numbers these relational differences are overshadowed by the differences that exist in total hippocampal size. Limits to the growth of individual cell populations independent of the growth of functionally related brain areas fit with the concerted view of brain development where developmental constrains cause coordinated changes in size (Finlay and Darlington, 1995; Finlay et al., 2001). A similar dynamic between evolutionary constraints and a complete structure can be seen in other structures of the brain (Gómez-Robles et al., 2014) or neurocranium (Mitteroecker and Bookstein, 2008). Considering the tight functional linkage between hippocampal principal cells, which has been equated with tight constraints on independent phylogenetic development (Whiting and Barton, 2003; Gómez-Robles et al., 2014), it is notable that we are still able to see strong separation of species based on convergence and divergence. Finding these relationships also illustrates the suitability of this data to study possible relationship between the different hippocampal regions and ecological and life-history variables.

Within the rodent group, the only exceptional species are the mole-rats that have unusually large CA3 cell numbers (discussed below). Interesting and perhaps reassuring is that quantitative relations in the common laboratory rodents: C57BL/6 mice, DBA mice, Wistar rats, and Sprague Dawley rats (brown rat) are at the center of the rodent cluster and, as far as quantitative relations are concerned, can be considered representative of rodents.

A known outlier species is the Sengi, which in size and habitat is similar to a rodent but shares quantitative relations closer to those of primates and the domesticated pig (Slomianka et al., 2013). Of the three primate species included in this study, the two monkeys share similar quantitative relations and, together with two additional data points from the literature (Leverenz et al., 1999; Jabès et al., 2011), provide further evidence for a quantitatively distinct monkey hippocampus. Humans are separated from all other species on account of a relatively large CA1, fitting with previous assessments of the human hippocampus (Stephan, 1983; Seress, 1988). However, CA1 has a rather low impact on overall species separation. What separates humans from other primates is less an exceptionally large CA1 but an exceptionally small hilus.

We feel that the number of species included does warrant the identification of a rodent cluster separate from other mammals in this study. The number of species in other taxonomic groups is however not sufficient to determine if quantitative relations in the hippocampus will be able to differentiate between these groups.

### Convergence and divergence

Primates, including humans, cluster tighter when we look at the relationship between connected areas by mapping the convergence and divergence in cell numbers. They share similar degrees of convergence of CA3 to CA1 cells, demonstrating that individual cell populations can differ to a large degree between species, while stable convergence/divergence relationships are retained in phylogenetically related species.

An observation relevant to current ideas about intrahippocampal information processing is the degree of convergence and divergence between the number of neurons in CA3 and CA1 in the three mole-rat species. Treves and Rolls (1994) have put forward the idea that a robust and noise-free down-stream transmission of information can be achieved if each CA1 neuron has to code less information compared to the CA3 neurons. Computational analysis of the information carrying capacity of the Schaffer collaterals show that an expansion rate of two, i.e., two CA1 neurons for every CA3 neuron, allows information to be passed without a significant loss. Increasing the expansion rate above two only leads to limited gains, while an expansion rate below one results in a rapid deterioration of information transfer between CA3 and CA1 (Treves, 1995; Schultz and Rolls, 1999). We observed four species with an “expansion” rate below one, of which the three most extremes cases are mole-rats. Mole-rats are known for having a relatively small overall brain size, which has been linked to reduced sensory input due to their strictly subterranean habitat (Harvey et al., 1980). With little information available on the hippocampal circuitry of mole-rats, one can only speculate if the limited sensory input puts a greater demand on the auto associative capabilities of the CA3 region or if it results in simpler CA1 ensemble codes requiring fewer cells. Also, it is not clear if and how this would be compatible with a loss of information in the transmission from the CA3 to the CA1.

As mentioned above, information transfer capability does not improve much at expansion rates above two and rapidly deteriorates at values below one. Yet, non-rodent species that we analyzed, except for marmoset monkeys, exceed the upper value, with humans reaching just above four. As pointed out by Schultz and Rolls (1999), these relations would allow CA3 cells to serve not only one but multiple segregated information streams. Quantitative observations relate well to the recent definition of subsets of CA1 pyramidal cells based on overlapping cytoarchitectural, neurochemical, and connectional criteria (Deguchi et al., 2011; Slomianka et al., 2011; Lee et al., 2014), physiological characteristics (Mizuseki et al., 2011; Hongo et al., 2015; Valero et al., 2015) and gene-expression data (Thompson et al., 2008; Dong et al., 2009; Zeisel et al., 2015). In the non-rodent CA1, quantitative relations would allow streams to be represented by non-overlapping cell populations that each can be served by the entire CA3 population. In rodents, in which most of the data pertaining to information streams were generated, the developmental matching of interconnected CA3 and CA1 cells (Deguchi et al., 2011) may prevent the deterioration predicted by theoretical models. Clear answers on how excessive redundancies or information deterioration are avoided will require refinements of the anatomical data and/or theoretical models.

### Do hilar cells punch above their weight?

The results of the present study suggest that changes in hilar cell numbers are associated with many taxonomic shifts. While the definitions of the principal cell populations appear rather straight forward in the rodents, this is not the case for non-rodents species with a reflected blade of CA3 (Figure 3D, number 2). The reflected blade of CA3 has also been named CA3h (Lim et al., 1997; Ding and Van Hoesen, 2015), avoiding the ambiguity associated with the heterogeneously defined alternative term CA4 (e.g., Lorente De Nó, 1934; Rosene and Van Hoesen, 1987). In all the sources that we have used and in the non-rodent species that we assessed ourselves, CA3h cells have been included in the estimates of hilar cell numbers (Figure 3D, number 2 and 3). In our case and most likely in the sources as well, this decision was made because a reliable border between CA3h and the remainder of CA3 is far easier to define than the border between CA3h and the hilar polymorphic cell layer that contains the bulk of the “proper” hilar cells. This must raise the question how an apparently arbitrary, technical border does impact on the interpretation of the outcomes. Some observations in our dataset suggest that the impact may be limited. First, hilar and CA3 cells differentiate between rodents in the same manner (similar strength and opposite directions) as they do between rodents and the remaining species. Second, hilar cells also differentiate species within the group that is characterized by inclusion of CA3h in hilar cell number estimates. Similar separations despite different definitions suggest that there is more to including CA3h cells in hilar estimates than technical reasons.

It would be difficult to argue a CA3 pyramid into being a hilar polymorphic cell, because of their distinct morphologies (Amaral, 1978; Buckmaster and Amaral, 2001), connectivities (Blackstad, 1956), electrophysiological properties (Scharfman, 1993; Buckmaster and Amaral, 2001) and development (Li et al., 2008). On the other hand, some of the ideas about these subfields have changed. The unidirectionality of information flow from the dentate gyrus to CA3 and the assignment of distinct functions to either the dentate gyrus (pattern separation) or CA3 (pattern completion) have softened enough to allow for bidirectional functional interactions between the dentate gyrus and proximal CA3 cells. Backprojections from CA3 to the dentate gyrus (reviewed in Scharfman, 2007) in the form of axon collaterals of proximal CA3 pyramidal cells to both the hilus (Ishizuka et al., 1990; Li et al., 1994) and deep dentate molecular layer (Li et al., 1994; Buckmaster and Amaral, 2001) provide feedback to hilar mossy cells, interneurons and granule cells (Scharfman, 1994, 1996; Kneisler and Dingledine, 1995). Notably, CA3 pyramidal cells extending dendrites into the dentate molecular layer have been found in CA3h of primates (Lim et al., 1997; Buckmaster and Amaral, 2001; Buckmaster, 2005). The suggestion that backprojections may provide a mechanism for CA3 to influence pattern separation (Myers and Scharfman, 2009) has been tested and used in computational models of CA3-dentate interactions (Myers and Scharfman, 2011; Petrantonakis and Poirazi, 2015), in which backprojections improve pattern separation in CA3. Consistent with these models, studies on the functional differentiation along the proximal to distal axis of CA3 found an emphasis on pattern completion in distal CA3 and on pattern separation in proximal CA3 (Lee et al., 2015; Lu et al., 2015), and both studies argue for a tight functional integration of proximal CA3 and the dentate gyrus in pattern separation. Functionally, this integration is also reflected in similar behavioral deficits following proximal CA3 and dentate lesions (Hunsaker et al., 2008) and *arc* expression in proximal as compared to distal CA3 pyramids in relation to pattern separation demands of changing environments (Marrone et al., 2014).

Therefore, it appears that, instead of hilar cells punching above their weight, it is more likely the interaction between proximal CA3 and the dentate and, consequently, the number of CA3 cells involved in this interaction that is a strong taxonomic separator between species. This idea would predict a much sharper functional differentiation between proximal and distal pyramidal cells in species in which proximal CA3 pyramids form a distinct CA3h. This idea could be tested in guinea pigs or rabbits, which both possess a well-defined CA3h (Geneser-Jensen, 1973; Geneser, 1987; Buckmaster et al., 1994). Lastly, it should be considered if a CA3h is indeed absent in rodents. We have previously discussed the possibility that cytoarchitectural changes in the temporal rodent hippocampus, in which backprojections are also stronger than septally (Li et al., 1994), suggest the presence of a CA3h (Slomianka et al., 2013). Lorente De Nó (1934) did identify a CA3h (the first reflected blade of his CA4; his Figure 2) in the mouse using the hippocampus temporal to the appearance of the lateral entorhinal cortex in his illustration.

### Neurogenesis as a separating factor

The stability of hippocampal principal cell numbers contrasts with an exponential age-related decline in AHN in all mammals that have been investigated (Ben Abdallah et al., 2010; Amrein et al., 2011). To allow for comparison of cell numbers, neurogenesis related cell counts were extrapolated to a common age of 3 month, which is the closes age for the majority of the rodents in our data set. To test the overall robustness of the estimates, additional analyses (testing different slopes for the decline of AHN and excluding mole-rats in which the error may be largest) were performed. Invariably and despite the small sizes of the cell populations representing AHN, neurogenesis is a major contributor to the separation of species in the rodent cluster. In mice, cell proliferation (marked by Ki67) and neuronal differentiation (marked by DCX) represent distinct stages of AHN, that can be differentially regulated to adjust the number of newly formed young neurons (van Praag et al., 1999; Kronenberg et al., 2003) and their maturation (Plümpe et al., 2006). The selective regulation is also reflected in our analysis in that DCX and Ki67 are independent separating factors, indicating that species use different strategies in the regulation of proliferation vs. survival and differentiation of new-born neurons. Differences in the regulation of AHN have been observed between and within taxonomic units before. Red foxes (Amrein and Slomianka, 2010) and non-human primates (Ngwenya et al., 2006, 2008, 2015; Kohler et al., 2011; Amrein et al., 2015) show a prolonged maturation phase of new-born neurons compared to rodents. Within rodents, habitat variability can be associated with different numbers of young neurons despite similar numbers of proliferating cells (Cavegn et al., 2013). Unexpectedly, when neurogenesis is added to the comparison, of all principal cell populations only hilar cells retain their power to separate between rodent species. Adult neurogenesis may provide an ontogenetic mechanism to adapt faster to changes in the ecological niche compared to the phylogenetic time scales that mediate changes in the principal hippocampal cell populations.

Without age-series for all the species that are included in this study, one remaining caveat will always be the possibility that it is not the number of new-born neurons at a particular age that is different between species, but rather the rate at which neurogenesis declines in a particular species. However, this does not change the separating power of AHN in the correspondence analyses and the subsequent interpretation of the data. In this scenario, it would be the differences in the rates of decline that serve as the plastic mechanism to adapt to specific ecological niches.

### Perspectives

Changes in the composition of the hippocampus in terms of the size of its' principal cell populations, the degrees of convergence and divergence of interconnected cell populations and adult neurogenic cell populations separate species groups at multiple taxonomic ranks. The value of this information lies in the provision of data points that may be useful in the computational modeling of hippocampal function and the issues raised in the contexts of the emerging concepts of hippocampal information streams and the functional differentiation of the CA3/dentate network. We have mostly abstained from speculations about the functional significance of these differences at the species level, which, more often than not, are difficult to substantiate and remain anecdotal. Provided that the species sample is large enough, statistical methods have become available that allow the detection of phylogenetic signals in the character distribution across a species sample (Pagel, 1999; Blomberg and Garland, 2002). Rank-ordering traits according to phylogenetic stability would, e.g., be one rational way to also rank them as targets of translational efforts. Also, large databases have been generated that define the life histories of species and the biotic and abiotic factors that characterize the niches that they occupy (Jones et al., 2009; Botero et al., 2014). Incorporating phylogenetic information (Freckleton et al., 2002), it has become possible to statistically associate ecological parameters with brain traits (Hutcheon et al., 2002; Finlay et al., 2014; Weisbecker et al., 2015), and we are currently extending our species sample to allow these techniques to be applied to the hippocampal cell populations.

## Author contributions

All authors listed, have made substantial, direct and intellectual contribution to the work, and approved it for publication.

## Funding

Swiss National Science Foundation (SNSF), 31003A_141244/1

### Conflict of interest statement

The authors declare that the research was conducted in the absence of any commercial or financial relationships that could be construed as a potential conflict of interest.
